# Frequency of Complete Remission With R-CHOP Therapy in Patients With Diffuse Large B Cell Lymphoma

**DOI:** 10.7759/cureus.57368

**Published:** 2024-04-01

**Authors:** Sani U Hassan, Shah Hussain, Mahnoor Fakhar, Azaz Ahmad, Fakeeda Durrani

**Affiliations:** 1 Department of Medical Oncology, Hayatabad Medical Complex Peshawar, Peshawar, PAK; 2 Department of Cardiology, Peshawar Institute of Cardiology, Peshawar, PAK; 3 Department of Medical Oncology, Fauji Foundation Hospital, Rawalpindi, PAK

**Keywords:** prognostic markers, r-chop therapy, complete remission, non-hodgkin lymphoma, dlbcl

## Abstract

Background

Diffuse large B-cell lymphoma (DLBCL) exhibits notable heterogeneity in clinical presentations and treatment responses, posing challenges in predicting outcomes and tailoring therapeutic strategies for affected patients. Despite advancements in molecular subtyping and prognostic assessment, uncertainties persist regarding the optimal management of DLBCL, highlighting the need for localized investigations to better understand treatment responses and outcomes within specific patient populations.

Objective

To assess the frequency of complete remission (CR) in diffuse large B-cell lymphoma (DLBCL) patients undergoing first-line rituximab, cyclophosphamide, doxorubicin, vincristine, and prednisolone (R-CHOP) therapy within a specific adult population.

Material and methods

This descriptive study was conducted within the Department of Oncology Hayatabad Medical Complex, Peshawar, Pakistan from August 8, 2022, to April 8, 2023. The study included newly diagnosed DLBCL patients aged 20-70 years, excluding those who had received prior treatment. There were 55 (57.9%) males and 40 (42.1%) females. Data on demographic characteristics, disease duration, and CR outcomes were collected using a predefined data collection form.

Results

The majority of patients (80, 84.2%) achieved CR following R-CHOP therapy. In terms of age distribution, 43 (45.3%) patients were aged ≤45 years, while the remaining belonged to the >45 years age group. The duration of the disease was ≤ 3 months in 60 (63.2%) cases, whereas it exceeded three months in 35 (36.8%) cases. With regards to BMI classification, nine (9.5%) patients had a BMI < 18.5 kg/m^2^, 49 (51.6%) fell within the range of 18.5-24.9 kg/m^2^, and the remaining 37 (38.9%) patients had a BMI between 25-30 kg/m^2^.

Conclusion

Diffuse large B-cell lymphoma (DLBCL) remains a heterogeneous disease entity with variable clinical outcomes. While R-CHOP therapy demonstrates promising efficacy in achieving CR, concerns regarding late adverse effects persist. Addressing these challenges requires continued research efforts to validate novel prognostic markers and develop alternative treatment approaches, ultimately improving patient outcomes and reducing the global burden of DLBCL.

## Introduction

Lymphomas represent a heterogeneous group of cancers arising from uncontrolled proliferation of lymphatic system cells, with lymphoid origin encompassing B-cell, T-cell, natural killer (NK)-cell, and Hodgkin lymphomas. Lymphomas, classified into various subtypes by the World Health Organization (WHO), demonstrate diverse global distributions [1]. Non-Hodgkin lymphoma (NHL) predominates, representing approximately 90% of cases, with Hodgkin lymphoma (HL) comprising the remaining 10% [2,3]. Despite recent advancements in understanding lymphogenesis at the molecular level, comprehensive knowledge regarding lymphoma subtype distribution remains limited, owing to historical treatment approaches that viewed lymphoma as a singular disease rather than a diverse entity of distinct neoplasms [4,5].

The efficacy of first-line rituximab, cyclophosphamide, doxorubicin, vincristine, and prednisolone (R-CHOP) therapy in achieving complete remission (CR) surpasses 75% among patients completing the standard six cycles [6,7]. Notably, those achieving CR post-R-CHOP demonstrate favorable survival outcomes, with a low risk of relapse within the initial five years, particularly among those maintaining a progression-free state for two years post-treatment evaluation. This suggests potential curative effects in the majority of diffuse large B-cell lymphoma (DLBCL) patients attaining first CR, as evidenced by mortality rates akin to the general population within a few years post-diagnosis [8,9].

Despite the promising outcomes associated with R-CHOP therapy, concerns persist regarding late adverse effects, notably secondary malignancies and cardiotoxicity, impacting DLBCL survivors during their extended lifespans [10]. Notably, data pertaining to the frequency of remission in DLBCL patients following R-CHOP treatment are variable across populations, underscoring the need for localized investigations to elucidate the extent of remission magnitude. Thus, this study aims to ascertain the prevalence of remission in DLBCL patients receiving R-CHOP therapy within our adult population. By addressing this gap in current literature, our findings will inform local healthcare providers, fostering awareness of the clinical landscape and guiding future research endeavors and therapeutic strategies.

## Materials and methods

This descriptive study was conducted within the Department of Oncology at Hayatabad Medical Complex, Peshawar, from August 2022 to April 2023. The study aimed to evaluate the frequency of complete remission (CR) in newly diagnosed DLBCL patients undergoing first-line R-CHOP therapy within a specific adult population.

The study included: newly diagnosed patients of DLBCL, age between 20-70 years, both genders and had not received prior treatment. Patients who had undergone any form of treatment prior to the initiation of the study were excluded from participation. Utilizing the WHO formula for sample size determination, a sample size of 95 is computed. This estimation is based on an anticipated CR proportion of 86% [11] among DLBCL patients receiving R-CHOP therapy, a 95% confidence level, and a 7% absolute precision.

Data on demographic characteristics, including age, gender, weight, height, body mass index (BMI), and disease duration, were collected using a predefined data collection form. Disease duration was defined as the time from initial diagnosis to presentation at the medical facility. Anthropometric measurements such as weight and height were obtained to calculate BMI. Additionally, information on CR outcomes following six cycles of R-CHOP therapy was recorded for each patient.

Descriptive statistics were employed to provide a comprehensive summary of both the demographic and clinical attributes observed within the study cohort, including means, standard deviations, frequencies, and percentages. The frequency of CR following R-CHOP therapy was reported as the primary outcome. Stratification analyses were performed to explore potential associations between demographic variables (age, gender, BMI) and CR rates. IBM SPSS 23.0 (IBM Corp., Armonk, NY, USA) was used for statistical analysis. Categorical variables were subjected to Chi-square tests for comparison, with statistical significance determined at p ≤ 0.05.

The study protocol received approval from the Research and Ethics Committee of Hayatabad Medical Complex, Peshawar, Pakistan, under reference number 495/HEC/B&PSC/2021. Informed written consent was obtained from all participants or their legal guardians after a thorough explanation of the research objectives and potential implications. The study adhered to the principles outlined in the Declaration of Helsinki and complied with other pertinent regulatory requirements, ensuring ethical standards and maintaining patient confidentiality.

To ensure the accuracy and reliability of the data collected, regular quality checks were performed throughout the study period. Data entry was double-checked, and any discrepancies or missing information were promptly addressed and resolved. Additionally, patient medical records were cross-referenced to verify the completeness and consistency of the data recorded.

## Results

Among total 95 patients, there were 55 (57.9%) males and 40 (42.1%) females. Age ranged between 20-70 years, with a mean of 46.4 ± 9.3 years. Mean weight, height, and BMI were recorded as 62.5 ± 11.3 kg, 171.4 ± 9.2 cm, and 21.7 ± 4 kg/m^2^, respectively. The mean duration of the disease at presentation was 2.8 ± 0.3 months (Table 1).

**Table 1:** Demographic and other characteristics (mean±SD)

Characteristics	Mean ± SD
Age	46.4±9.3 years
Weight	62.5±11.3 kg
Height	171.4±9.2 cm
BMI	21.7±4 kg/m^2^
Duration	2.8±0.3 months

In terms of age distribution, 43 (45.3%) patients were aged ≤45 years, while the remaining belonged to the >45 years age group. The duration of the disease was ≤3 months in 60 (63.2%) cases, whereas it exceeded three months in 35 (36.8%) cases. With regards to BMI classification, nine (9.5%) patients had a BMI < 18.5 kg/m^2^, 49 (51.6%) fell within the range of 18.5-24.9 kg/m^2^, and the remaining 37 (38.9%) patients had a BMI between 25-30 kg/m^2^ (Table 2).

**Table 2:** Frequency (%) of demographic and other characteristics

Characteristics	Frequency	Percentage
Male	55	57.9%
Female	40	42.1%
≤45 years age group	43	45.3%
> 45 years age group	52	54.7%
≤ 3 months	60	63.2%
> 3 months	35	36.8%
BMI		
< 18.5 kg/m^2^	9	9.5%
18.5-24.9 kg/m^2^	49	51.6%
25-30 kg/m^2^	37	38.9%

Complete remission was documented in 80 (84.2%) cases of the total cohort after six cycles of R-CHOP therapy. Upon gender stratification, complete remission rates were observed in 48 (87.3%) cases of male patients and 32 (80%) cases of female patients. Further stratification by gender revealed complete remission rates of 37 (86%) cases in individuals aged ≤45 years and 43 (82.7%) cases in those aged >45 years.

Analysis based on BMI categories demonstrated complete remission rates of six (66.7%) cases in individuals with a BMI level of < 18.5 kg/m^2^, 40 (81.6%) cases in those with a BMI between 18.5-24.9 kg/m^2^, and 19 (51.3%) cases in individuals with a BMI between 25-30 kg/m^2^, p=0.002 (Table 3).

**Table 3:** Stratification of complete remission with respect to gender, age and BMI

Variable	Frequency	Percentage	P value
Complete remission	80	84.2%	
Gender			
Male	48	87.3%	0.087
Female	32	80%	
Age			
≤45 years	37	86%	0.888
> 45 years	43	82.7%	
BMI			
< 18.5 kg/m^2^	6	66.7%	0.002
18.5-24.9 kg/m^2^	40	81.6%	
25-30 kg/m^2^	19	51.3%	

## Discussion

Diffuse large B-cell lymphoma (DLBCL) represents a significant proportion of newly diagnosed non-Hodgkin lymphoma (NHL) cases in developed nations, comprising approximately 31% to 40% of all NHL diagnoses [12]. Subtyping DLBCL based on its origin into germinal center B-cell (GCB) and non-GCB subtypes, as well as further categorization into GCB, activated B-cell (ABC), and unclassified (UNC) subtypes via gene expression profiling, has provided valuable insights into the heterogeneity of this disease [13]. Numerous studies have consistently demonstrated that patients with non-GCB and ABC subtypes exhibit poorer survival outcomes compared to those with the GCB subtype [14]. This molecular classification has enabled more personalized treatment strategies, with emerging evidence suggesting differential responses to various therapeutic approaches based on DLBCL subtype [15].

In addition to molecular subtyping, several clinical characteristics have emerged as prognostic indicators in DLBCL. The International Prognostic Index (IPI), initially developed as a prognostic tool for NHL, including DLBCL, incorporates factors such as age, disease stage, extranodal involvement, performance status, and serum lactate dehydrogenase (LDH) levels. While the IPI has historically served as a valuable prognostic tool, its predictive accuracy has been challenged in the era of rituximab-based therapy [16]. To address this limitation, the National Comprehensive Cancer Network-IPI (NCCN-IPI) was introduced, incorporating additional factors such as absolute lymphocyte count and presence of bulky disease, to better stratify patients receiving rituximab-containing regimens [17].

Despite these advancements, challenges remain in accurately predicting outcomes and tailoring treatment strategies for DLBCL patients. For instance, novel prognostic indices, such as the Grupo Español de Linfomas/Trasplante Autólogo de Médula Ósea - International Prognostic Index (GELTAMO-IPI), which integrates serum β2-microglobulin levels with National Comprehensive Cancer Network International Prognostic Index (NCCN-IPI), have shown promise in enhancing prognostic accuracy [18]. However, their widespread implementation is hindered by data limitations and practical constraints. Moreover, while rituximab-based regimens have significantly improved treatment response and survival in DLBCL, disparities in access to rituximab and its optimal dosing persist, particularly in resource-limited settings [19]. This highlights the need for equitable access to effective therapies and the development of alternative treatment strategies for DLBCL patients worldwide.

Our study showed that 80 out of 95 patients (84.2%) achieved CR after six cycles of R-CHOP therapy, this aligns with findings from prior research that reported CR rates ranging from 60% to 90% in patients with DLBCL treated with R-CHOP [20,21]. The median age of our patients was 46.41 years, which is lower than the median age of 70 years reported in most studies of DLBCL [22]. This may reflect the younger population of our region. We found that age, gender, and disease duration were not significantly associated with CR in our study. However, we observed that patients with a lower BMI had a higher frequency of CR than those with a higher BMI (66.7 & 81.4% vs 51.3%, p = 0.002). This finding is in contrast to some studies that reported a positive correlation between BMI and CR in patients with DLBCL [23], but in agreement with others that found no association or a negative correlation [24]. The possible mechanisms underlying the relationship between BMI and CR are not clear, but may involve the pharmacokinetics of R-CHOP drugs, the inflammatory status of obese patients, or the tumor microenvironment.

Limitations

The limited sample size, descriptive study design and single-center nature of the study may restrict the generalizability of the findings to broader patient populations. Furthermore, incomplete data on novel prognostic markers, such as serum β2-microglobulin levels, hindered comprehensive analysis and may have influenced the observed associations. Future studies employing prospective, multicenter designs with larger sample sizes and comprehensive data collection are warranted to address these limitations and provide further insights into the management of DLBCL.

## Conclusions

Our study underscores the effectiveness of R-CHOP therapy in achieving complete remission (CR) among diffuse large B-cell lymphoma (DLBCL) patients, with a significant proportion experiencing favorable outcomes. While age, gender, and disease duration did not significantly impact CR rates, our findings suggest a potential association between lower BMI levels and higher CR rates, warranting further investigation. Future studies should focus on validating novel prognostic markers, developing alternative treatment approaches with improved safety profiles, ensuring equitable access to effective therapies, and collecting comprehensive data to optimize patient care and outcomes in DLBCL. These efforts are essential for advancing our understanding of DLBCL and tailoring personalized treatment strategies to improve patient outcomes and reduce the global burden of this disease.
